# Towards a comprehensive research and development plan to support the control, elimination and eradication of neglected tropical diseases

**DOI:** 10.1093/trstmh/traa114

**Published:** 2020-11-11

**Authors:** David Mabey, Ellen Agler, John H Amuasi, Leda Hernandez, T Déirdre Hollingsworth, Peter J Hotez, Patrick J Lammie, Mwelecele N Malecela, Sultani H Matendechero, Eric Ottesen, Richard O Phillips, John C Reeder, Célia Landmann Szwarcwald, Joseph P Shott, Anthony W Solomon, Andrew Steer, Soumya Swaminathan

**Affiliations:** Clinical Research Department, London School of Hygiene & Tropical Medicine, London WC1E 7HT, UK; The END Fund, New York, NY 10016, USA; African Research Network for Neglected Tropical Diseases, Kumasi AK-039-5028, Ghana; Department of Health, Infectious Disease Office, National Center for Disease Prevention and Control, Manila 1003, Philippines; Big Data Institute, Li Ka Shing Centre for Health Information and Discovery, University of Oxford, Oxford OX3 7LF, UK; Departments of Pediatrics and Molecular Virology & Microbiology, Texas Children's Hospital Center for Vaccine Development, National School of Tropical Medicine, Baylor College of Medicine, Houston, TX 77030s, USA; Hagler Institute for Advanced Study at Texas A & M University, College Station, TX 77843, USA; Department of Biology, Baylor University, Waco, TX 76706, USA; James A. Baker III Institute of Public Policy, Rice University, Houston, TX 77005, USA; Scowcroft Institute of International Affairs, Bush School of Government and Public Service, Texas A & M University, College Station, TX 77845, USA; Neglected Tropical Diseases Support Center, Task Force for Global Health, Decatur, GA 30030, USA; Department of Control of Neglected Tropical Diseases, WHO 1211, Geneva, Switzerland; Division of Communicable Disease Prevention and Control, Neglected Tropical Diseases Unit, Ministry of Health, Nairobi, Kenya; Neglected Tropical Diseases Support Center, Task Force for Global Health, Decatur, GA 30030, USA; Kumasi Center for Collaborative Research in Tropical Medicine, Kwame Nkrumah University of Science and Technology, Kumasi AK-039-5028, Ghana; UNICEF, UNDP, World Bank, WHO Special Programme for Research and Training in Tropical Disease (TDR), 1211 Geneva 21040-900, Switzerland; Instituto de Comunicação e Informação Científica e Tecnológica em Saúde, Fundação Instituto Oswaldo Cruz, Rio de Janeiro, RJ, Brasil; Division of Neglected Tropical Diseases, Office of Infectious Diseases, Bureau for Global Health, USAID, Washington, DC 20004, USA; Department of Control of Neglected Tropical Diseases, WHO 1211, Geneva, Switzerland; Department of Paediatrics, University of Melbourne, Melbourne, Victoria 3010, Australia; Department of General Medicine, Royal Children's Hospital, Melbourne, Victoria 3052, Australia; Tropical Diseases Research Group, Murdoch Children's Research Institute, Melbourne, Victoria 3052, Australia; Science Division, WHO, Geneva 1211, Switzerland

## Abstract

To maximise the likelihood of success, global health programmes need repeated, honest appraisal of their own weaknesses, with research undertaken to address any identified gaps. There is still much to be learned to optimise work against neglected tropical diseases. To facilitate that learning, a comprehensive research and development plan is required. Here, we discuss how such a plan might be developed.

Impressive progress has been made against neglected tropical diseases (NTDs) since the publication of the first NTD road map^1^ and the London Declaration on NTDs^2^ in 2012. At least one NTD has been eliminated from 40 countries, territories or areas, 500 million fewer people require interventions against NTDs^3^ and effective oral treatment regimens have become available for Buruli ulcer, yaws and stage 2 human African trypanosomiasis due to *Trypanosoma gambiense*.^4-6^ The 2021–2030 NTD road map,^3^ which is awaiting World Health Assembly endorsement, seeks to build upon this progress by setting or reaffirming a number of ambitious targets, including elimination of at least one NTD from 100 countries and global eradication of yaws and dracunculiasis.

As we pursue these targets over the next 10 years it will be important to learn from previous elimination and eradication programmes. The yaws eradication programme, launched by the WHO in 1952, was hugely successful, examining 460 million people, treating more than 50 million and reducing the global prevalence of yaws by >95%. However, as prevalence fell and transmission interruption was approached in many countries, funding became increasingly hard to obtain. Postelimination surveillance was not established and the disease subsequently returned.^7^

The endgame may be the most difficult part of an elimination programme. It can be frustrating. In the case of Guinea-worm disease, the number of new cases was reduced from more than 3 million per year in the 1980s to 28 in 2018, but in 2019, 54 human cases were reported, including one in Angola, a country that had only ever reported one previous human case.^8^ In Chad, nearly 1500 infected dogs were identified; to eliminate this reservoir, research is needed to understand how dogs are infected and how infection can be prevented.^9^ In the case of trachoma, where the definition of elimination is based on prevalence of physical signs, it has become increasingly difficult to train healthcare workers to identify cases as cases become less common; in addition, in some populations, the signs are not specific and many individuals have follicular conjunctivitis not due to trachoma. Recent research has focused on the potential use of serology to determine whether transmission of ocular *Chlamydia trachomatis* infection has been reduced sufficiently to make future trachoma-related blindness unlikely.^10^

To have confidence that elimination targets have been met, large numbers of people (and, in the case of some vector-borne diseases, large numbers of vectors) must be tested.^11-13^ The cost of such efforts is not insignificant.^14^ New, high-throughput diagnostic tests are required, some of which will need to work efficiently at point-of-human-care or be useful for testing intermediate or reservoir hosts. Tests must be highly specific (to avoid confusion due to false positives) and sensitive enough to detect the few remaining cases. Target product profiles for diagnostics for mapping and monitoring some NTDs, and for postelimination surveillance, were published in 2012^15^ but need to be updated, and should facilitate development of integrated multi-disease testing platforms to increase the cost-effectiveness of monitoring. Development of updated diagnostic target product profiles is now being undertaken by WHO's recently established NTD Diagnostics Technical Advisory Group. Political, financial and technical investment to fully capacitate laboratories will be essential for undertaking the requisite studies plus routine testing.

In the case of lymphatic filariasis, for example, the traditional diagnostic method of identifying microfilariae in blood samples was recognised as imperfect, since in many places it requires night-time blood collection (because of the nocturnal periodicity of microfilariae) and subsequent painstaking microscopy. A point-of-care serological test for circulating filarial antigen (CFA) was therefore developed and is now used to validate elimination; the elimination target is a prevalence of CFA positivity of <2.0% in children aged 6–8 y, persisting for at least 4 y after cessation of mass treatment.

For some other NTDs, the endgame feels a long way away. Mycetoma, chromoblastomycosis and other deep mycoses were added to the WHO's NTD list in 2016; snakebite envenoming, and scabies and other ectoparasites, were added in 2017. Development of comprehensive packages of epidemiological assessment and public health intervention for these conditions is at an early stage.^16-19^ For yaws, the geographical extent of the endemic population and the required number of rounds of mass treatment to interrupt transmission are each far from certain,^20,21^ and both macrolide resistance and infection in non-human primates put eradication at risk. Due to multiple factors including population growth, enhanced travel, urbanisation and expansion in the range of the vector mosquitoes, the global incidence of dengue is increasing year on year.^22^ For soil-transmitted helminthiases and schistosomiasis, reinfection may be rapid after preventive chemotherapy applied alone; programmes may need other effective interventions to maximise their impact.^23,24^

Compounding these complexities is the local amplification of NTD transmission by multiple forces, including war and political collapse,^25^ urbanisation^26^ and shifting poverty. Climate change is also affecting the geographical distribution of multiple NTDs. Since 2020, the COVID-19 pandemic threatens the progress of NTD programmes through multiple mechanisms.

To help combat these challenges, we must face the reality that, in addition to better diagnostics, we urgently need new and improved NTD vaccines and drugs. The dramatic acceleration of work to generate analogous tools against COVID-19 is a clear signal that we could achieve similar progress in NTD science if we had the requisite political will and drive.^27^

A comprehensive research and development blueprint could help address these challenges by allowing scientists and donors to contribute in a more coordinated way. How would we, as an extended NTD community, develop such a blueprint and make it maximally effective for driving change?

The answer is likely to be a combination of actions and attitudes. The list includes: an awareness of history; synthesis and honest evaluation of existing evidence; modelling of current programme trajectories and probable future needs; frank conversations involving all relevant stakeholders; the recognition that effort has already been invested in curating research priorities for some diseases,^18,28-30^ forming possible starting points for compilation of a more comprehensive research agenda; hard work; collaboration; research capacity-building in NTD-endemic countries; and a willingness to review, reflect and change as things evolve.

The hard work has already started. The WHO has recognised the listing of identified NTD research priorities within its Global Observatory on Health research and development platform (https://www.who.int/research-observatory/en/) to be a Global Public Health Good, allowing internal resources to be devoted to the task. And it has commenced an internal review of existing lists of disease-specific research priorities, including those contained in the 2021–2030 roadmap. The weaknesses of existing lists include a lack of clarity on and consistency in the approach to their development, and differences in the structuring of prioritised questions across diseases, which could limit thinking about the integration of research and programmes.

Dedicated research to support the integration agenda is overdue.^31^ All elements of NTD programmes—including programme management, epidemiological assessment, disease control and evaluation—could potentially benefit. Integration could include linking co-endemic NTDs, better connecting NTD programmes with efforts against other communicable and non-communicable diseases, as well as joining forces with development programmes outwith the health sector.

Cross-cutting socioanthropological and health systems research is also needed to guide programmes on how and when to deliver new (and existing) tools to end-of-the-road communities in the most cost-effective way. Regardless of their quality, commodities do not deliver themselves. Implementation research is key to ensuring that the time and money invested in other research and programmatic elements are not wasted.

Consultation to gather input from the wider NTD community will be undertaken in 2020. In the altered world of the COVID-19 pandemic, convening an inclusive forum to undertake this process may be more straightforward than it might otherwise have been. If managed well, online meetings can be an equaliser, in which the strength of ideas matters more than stridency of speech, and the involvement of greater numbers of more diverse constituencies becomes possible with marked reductions in the cost of meeting attendance.

In the meantime, while a research and development blueprint is being developed and the questions it identifies are being answered, work to alleviate the suffering caused by NTDs should not stop. We already have many proven, cost-effective interventions. To this end, we should continue to strike a measured balance between expressing confidence in the valuable tools that we already have and the uncertainty elsewhere that will drive further progress. For that, skilled and credible advocates will be needed, as well as partners willing to hear a complicated tale.

## Data Availability

None.
